# Practical Notes and Formulæ

**Published:** 1885-08-20

**Authors:** 


### PRACTICAL NOTES AND FORMULÆ.

**Iris and Euonymus.**—I live in a latitude where chronic ague and masked intermittents are as common as children. After trying in vain quinia, arsenic, nux, and acetate of potash, I fell on the following:

℞. Tinct. iris ...................................... ʒj

Tinc. euonymus ...................................... ʒij

Water ...................................... ℥iv.

A teaspoonful three times per day till all is taken. I have never failed in a single instance. I use the same prescription in sub-acute rheumatism and neuralgia (chronic form) with flattering success. The iris is a splended alterative, and will cure anything from acne to the largest size of Job's comforters.

If any patient persist in having his liver "tapped," give him five-drop doses of tincture of iris every three hours till his bowels move. You can satisfy the most skeptical that calomel is not the only cholagogue.—*Ec. Med. Journal.*

**A Specific for Burns.**—The application of soda bicarb. to burns has never given any satisfaction to myself or my patients, and I am inclined to think that the only good that has ever resulted from its use has been obtained by smearing it thickly over the affected part; thereby excluding the air and simply acting as the coating of white lead and linseed oil so earnestly and justly advocated bv Prof. Gross. Such applications only act as mere covering. But if you will apply balsam copaiba freely over the next burn you have to treat, you will have a more correct idea of the meaning of the term "specific" than by any other means I know of. The squalling child will quiet down in a few minutes. You will return home paid, and the mother will bless the "bully doctor." The same balsam is equally specific to the cure of frozen parts, removing the pain and soreness in a few applications.—*Ec. Med. Journal.*

**Treatment for Carbuncles.**—Dr. Bulkley, of New York, recommends the following ointment as a dressing for carbuncles:

℞. Ext. ergot ...................................... f ʒij

Zinci. oxidi ...................................... ʒss

Ung. aq: rosæ ...................................... ℥ij

M.

In conjuction with this, he gives sulphide of calcium, one-quarter grain, every two hours, and a refrigerant tonic, containing diluted sulphuric acid and sulphate of iron.

**Atropine in Epilepsy.**—Dr. David advises the treatment of epilepsy by the simultaneous employment of atropine and the bromides of potassium and ammonium. For a period of six months, twenty grains of the bromide of ammonium, thrice daily. At the same time the patient is instructed to take a granule of one milligramme of sulphate of atropine morning and evening. At the end of six months the following pills are prescribed:

℞. Valerianate of zinc........................4 centigr.
Ext. of belladonna.......................6 milligr.
Arsenious acid........................2 milligr.
Extract of gentian........................q. s.

Two of these pills are taken daily during twelve months. Should the faintest symptoms of threatened occurrence of epilepsy appear, the treatment must be kept up for yet another twelve months.—*Lyon Medical Journal.*

**Diarrhœa Mixture.**—Dr. E. X. Veat, in N. E. Med. Monthly, says, I send for the benefit of the readers of the Monthly, the following formula for a diarrhœa mixture which I am in the habit of using largely in my practice, and with most excellent results:

℞. Extract of hæmatoxylin..........................ʒiv

Tinct. opii camph...........................℥j

Tinct. zingiberis..............................

Tinct. rhei co................................aa ℥ss.

M. Dose, a teaspoonful every two or three hours.

**Ringworm.**—

℞. Thymol..................................ʒj to ij

Chloroform..............................℥j

Olive oil..................................℥iij

M. The thymol destroys the fungus, the oil prevents irritation and rapid evaporation, while the chloroform facilitates the absorption of the active ingredients by acting on the sebaceous glands.—*Medical World.*

As a cheap prescription for chills, Dr. J. B. Johnson (Medical and Surgical Reporter) recommends the following:

℞. Sulphate of cinchonidia,
Sulphate of cinchona, } ..............aa grs. xx
Powd. purified chinoidine,
Powdered aloes..............................grs. x
Powdered sulphate of iron,
(ferri sulph. exs.), } ..............aa grs. xx
Powdered capsicum,
Syrup..................................q. s.

M. Divide into twenty-one pills. Sig. Dose, three pills every three hours.

These pills I have found to be not only efficacious in arresting the chills, but a most excellent tonic in giving tone to the general system after the chills have been arrested; and for this purpose I required my chill patients to continue them for a month or six weeks to prevent a lapse.—*Louisville Medi. Journal.*

